# A rare case of carpal boss lesion with an overlying ganglion cyst: case report and literature review

**DOI:** 10.1093/jscr/rjae287

**Published:** 2024-05-03

**Authors:** Ahmad Nasser Bo Eissa, Ahmed Khalid Almulhum, Mohammed Nooh Alsaeed, Ali Ahmed Buanq

**Affiliations:** Department of Orthopedics, Almoosa Specialist Hospital, AlAhsa, Hofuf, Dhahran Road, Alkhars District, Saudi Arabia; Department of Orthopedic Surgery, King Fahad Hufof Alhufayrah District, Saudi Arabia; Department of Orthopedic Surgery, King Fahad Hufof Alhufayrah District, Saudi Arabia; Department of Orthopedic Surgery, Bahrain Defense Force Hospital Military Hospital, Riffa, Southern Governate Road, Wadi Alsail District, Bahrain

**Keywords:** carpal boss, carpometacarpal bossing, wrist pain, hand surgery

## Abstract

Carpal boss is a bony protrusion on the dorsal aspect of wrist quadrangular joint. The exact cause and prevalence are not well understood. Most of the patients are asymptomatic, although some experience pain, bony prominence, restricted mobility, and joint instability in sever neglected cases. We are presenting a case of a 24-year-old male had chronic dorsal wrist pain with bony prominence appearance underwent surgical excision of a carpal boss lesion in concomitant with soft tissue ganglion cyst over the carpal boss after failed conservative management, resulting in significant symptom improvement and restored range of motion. Carpal boss lesion is a common condition that can be undiagnosed due to asymptomatic presentation or the presence of overlying soft tissue pathology as ganglion cyst. Although conservative treatment is helpful in some patients, most symptomatic carpal boss lesion patients eventually need surgical excision.

## Introduction

Carpal boss or carpometacarpal (CMC) bossing lesion is a bony protuberance on the dorsal aspect of the quadrangular joint of the wrist, which involves the base of the second and third metacarpal bones, the capitate, and the trapezoid [1]. The epidemiology of carpal boss lesions is not well understood. However, cadaveric studies have reported a prevalence up to 19% [2]. It affects the adults in their fourth decade of life in their dominant hand with no clear male or female preference. However, few studies showed a slight male predominance [3, 4]. The exact etiology of carpal boss is not well established yet. However, studies showed that it may occur due to degenerative osteophyte formation, presence of os-stylodium, and post-traumatically [4]. Clinically, most of the patients are asymptomatic and it is discovered accidentally upon X-rays. Although, the minor symptomatic patients usually present with pain, palpable swelling over the dorsum of the wrist, restricted mobility, and joint instability. These symptoms are linked to concomitant degenerative osteoarthritis, ganglion cyst, or an extensor tendon slipping over it [1, 3]. The treatment of carpal boss lesion is observation for asymptomatic patients. In contrast, non-steroidal anti-inflammatory medications, immobilization and splinting, physiotherapy is indicated as for symptomatic patients. Steroid injection and eventually surgical excision for refractory cases [5, 6].

## Case presentation

**Figure 1:**
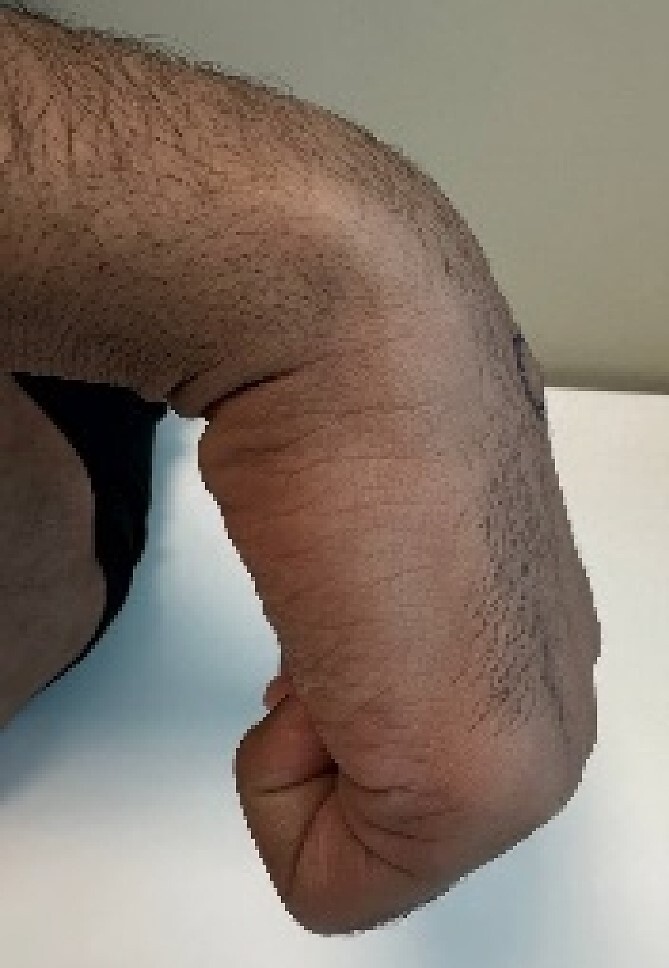
Pre-operative hand and wrist examination.

**Figure 2:**
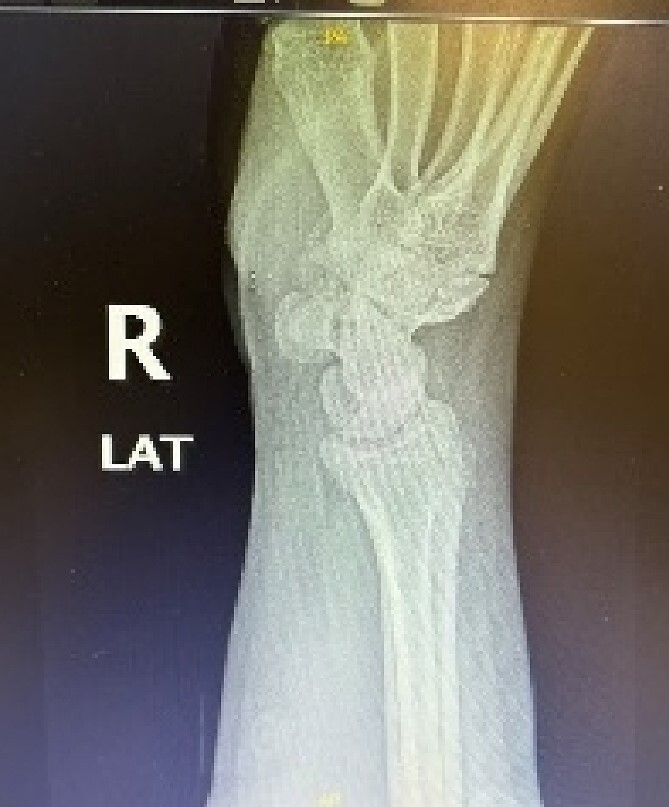
Hand and wrist x-rays pre-operatively.

**Figure 3:**
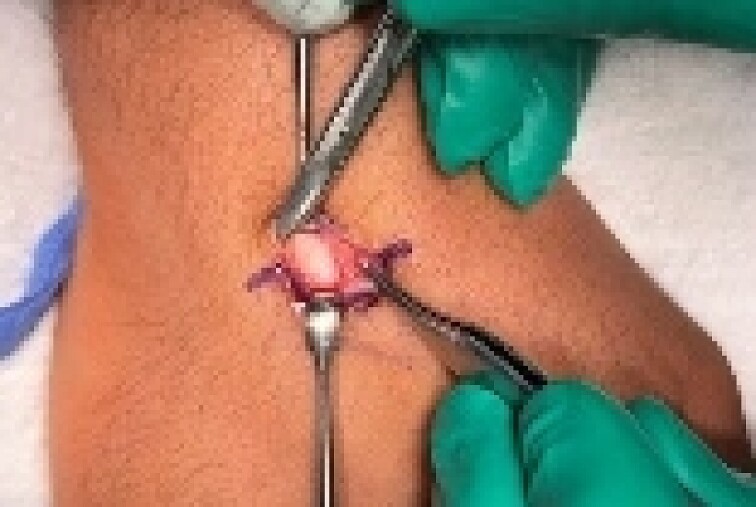
A dorsal approach of the quadrangular joint with excisional biopsy of the cystic lesion and bony lesion.

**Figure 4:**
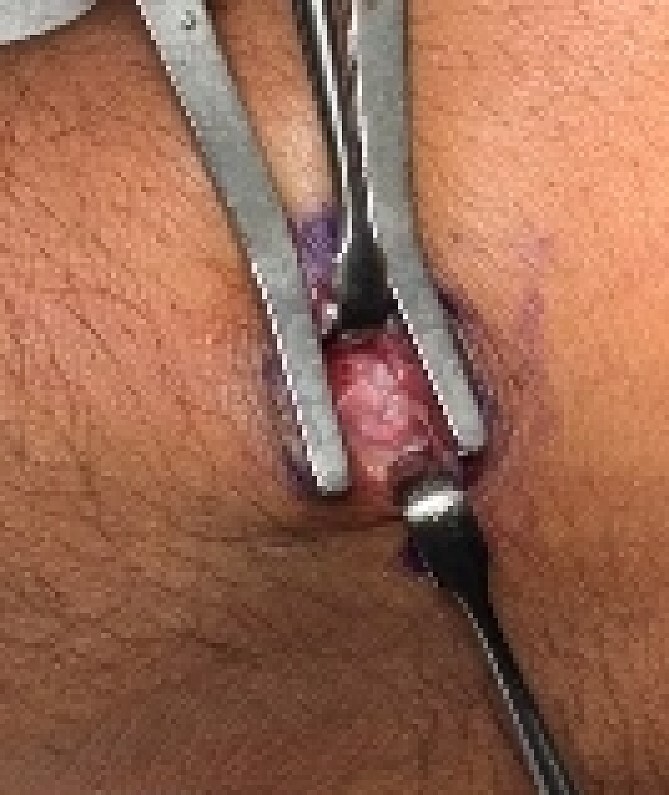
A dorsal approach of the quadrangular joint with excisional biopsy of the bony lesion.

**Figure 5:**
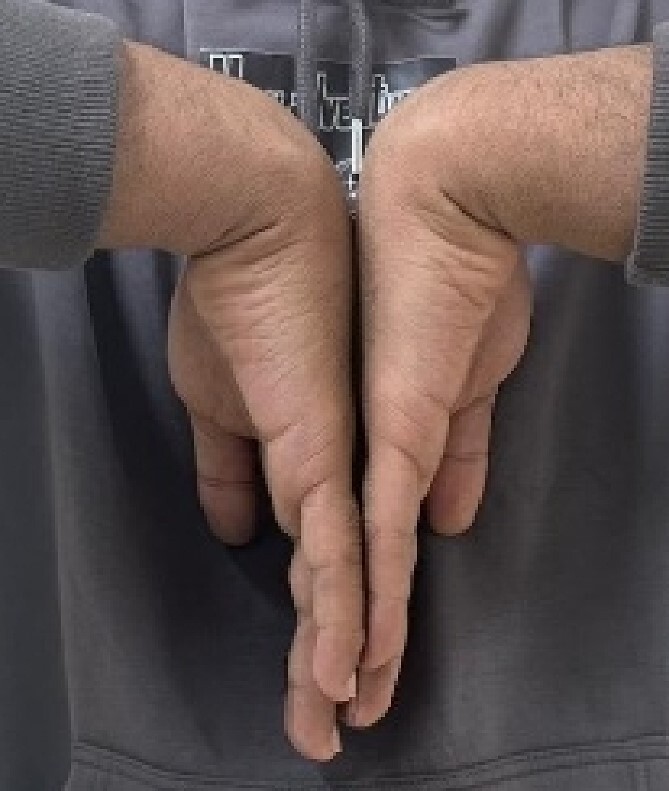
Hand and wrist range of motion 2 months post-operatively.

A 24-year-old male patient presented to the orthopedics clinic complaining of right-hand swelling for 2 years in the dorsal aspect of his hand described as ball like lesion. It started gradually over time without any triggering factors. However, in the last 6 months the patient has reported a mild on/off pain over the lesion that is dull aching aggravated with hand motion. However, the pain slightly declined with the use of analgesics. The pain was 3/10 in severity with some daily activity interruption with some range of motion (ROM) limitation. The patient had no constitutional, past medical, or surgical history with no significant family history. The patient has tried conservative management for 3 months but showed minimal improvement. Upon examination, there was right hand dorsal oval shaped swelling with no evidence of deformity, muscle wasting or overlying skin changes. On palpation, there was palpable lump noted over the second and third carpometacarpal joints ~1 cm in diameter that is well circumscribed, soft in consistency, and mobile. The lump is palpated over and surrounded by an underlying hard bony protuberance with mild tenderness (Fig. 1). The right hand and wrist range of motion showed 5–10 ° flexion and extension limitation of wrist motion with accompanying pain and second and third CMC joints with no affection of any other movements. Furthermore, the piano, distal radioulnar joint squeeze, Watson tests and neurovascular examination, extensor tendons examination were unremarkable. In the meantime, a ganglion cystic lesion was suspected with and underlying bony lesion. Meanwhile, his laboratory investigations were unremarkable. Hand and wrist X-rays were obtained and showed osteophyte formation in the base of the second and third metatarsal and distal aspect of the capitate and trapezoid with joint space narrowing consistent with carpal boss lesion (Fig. 2). Hand and Wrist Ultrasonography also performed showed well defined subcutaneous cystic lesion measuring about 7 × 2 mm consistent with ganglion cyst seen in between related bony prominence. Following that, the patient underwent an open excision of the carpal boss, and ganglion lesions. Intra-operatively, through the dorsal wrist approach over the quadrangular joint of the wrist, the retinaculum and extensor compartments were incised until soft oval ganglion cyst measuring about 0.5 cm in diameter was found and excised (Fig. 3). Upon further exposure, irregular shaped bony overgrowth ~1 cm in diameter was noticed underlying the cystic lesion (Fig. 4). The lesion and its surrounding sclerosis excised using wedge excision technique using osteotomes and ronjour while avoiding injuring the intra-articular cartilages (Fig. 4). Histopathology of the lesions revealed a cystic lesion with flbro-collagenous cyst wall with fragments of benign mature bone with overlying fibrous tissue, no mature Hyaline cartilage component is seen, and the medullary cavity contains fat and hematopoietic marrow. Postoperatively, the patient had no neurovascular compromise. X-rays were obtained and showed flattening of either sides of the quadrangular joint of the wrist with an adequate excision of the bony outgrowth. The patient was followed 2 weeks later in which stitches were removed and instructed on rehabilitation. Upon 2 months follow up, the patient had no complaints. Upon examination, there is no lesion that can be inspected or palpated with no tenderness with healed surgical scar. Also, the patient showed no pain and limitation of hand and wrist range of motion with intact extensor tendons and CMC ligamentous functions (Fig. 5). Six months postoperatively, the patient was symptoms free. The scar showed healthy tissue. Hand and Wrists examinations showed no limitation. X-rays showed no evidence of re-growth or degenerative changes. Eventually, the patient reported that his quality of life had improved significantly, especially in terms of pain, and range of motion with no disturbance in hand and wrist functions and he was very happy with the results. This case has been reported in line with the Surgical Case Report (SCARE 2020) [7].

## Discussion

A carpal boss lesion is an osseous outgrowth of the quadrangular joint of the wrist commonly involves the dominant hand with no clear male to female difference [1–4]. Various theories were raised about the etiology of the lesion formation which includes degenerative osteophyte formation, post-traumatic sequela, os styloideum, CMC joint coalition. However, the exact cause remains unclear yet [4]. Most of the patients are asymptomatic, and it is discovered either accidentally or as a cosmetic disorder. The degenerative presentation is the commonest, and the rarest is the congenital [4, 8]. A symptomatic patient usually presents with dorsal wrist joint swelling most visible during wrist flexion with concomitant pain that is provoked by repetitive activity and relived with rest or analgesia. Also, patients may have restrictions in hand and wrist ROM, but this is uncommon. These symptoms are attributed to osteoarthritic changes, overlying ganglion, bursitis, or extensor tendon irritation [2, 4, 9]. The mass is hard in consistency and immobile. However, the lesion might not be appreciated if an associated overlying soft tissue lesion is present, such as ganglion cyst, lipoma, or neuroma, which needs to be on the list of differentials and should not mask the suspicion of the carpal boss. However, hard bony lesions are also considered such as intraosseous ganglion, osteochondroma, and osteosarcomas [9–11]. In our case, we reported a carpal boss lesion that was misdiagnosed initially prior to radiological investigations due to the presence of an overlying ganglion cyst. Radiologic assessment of the carpal boss lesion involves plain radiographs of the wrist and/or hand with carpal boss view, and it is often sufficient. However, more specialized radiological investigations needed when radiographs are not diagnostic, to identify the exact cause, to look for any masking lesions, such as ganglion cysts, as in our patient [4, 9, 12]. Up to date, there is no consensus about the optimal treatment for the carpal boss [3, 4]. However, observation is the key for asymptomatic patients. In contrast, the initial treatment of symptomatic patients involves rest, Non-Steroidal Anti-Inflammatory Medications, a short period of immobilization in a splint, and hand physiotherapy. Currently, there is no gold standard surgical procedure for carpal bossing [6, 9]. Wedge excision is the most preferred choice as it involves an excision of the carpal boss with its surrounding sclerosis as far as undamaged cartilage is seen in all directions of the quadrangular joint [4, 9, 13–15]. It has a lower recurrence rate as compared to simple excision, which showed a failure rate ranging from 5% to 50% [15]. However, excessive resection, even with wide-wedge excision, can cause bony rebuilding or dorsal carpometacarpal ligament disruption, which in turn results in CMC joint instability. We presented an unusual case of a carpal boss lesion with an overlying ganglion cyst, which was misleading during examination and imaging. Following failed conservative treatment. An excision biopsy of the cyst and a wide-wedge excision were done for the carpal boss. Upon a 6-month follow-up, the patient showed no recurrence of the bump and symptoms with no associated complications.

## Conclusion

The case shows the importance of a comprehensive evaluation for patients with persistent dorsal hand pain which could be related to carpal boss lesions to provide the patient with the appropriate management plan. Surgical excision can be an effective treatment option for symptomatic patients who have failed conservative measures. Long-term follow-up is necessary to monitor for recurrence and ensure sustained improvement. However, further studies are needed to investigate the exact prevalence and etiology of carpal boss lesion.

## References

[1] Fiolle MJ . Le “carpe bossu”. Et Mem Soc Nat Chir 1931;57:545–7.

[2] Alemohammad AM , Nakamura K, El-Sheneway M, Viegas SF. Incidence of carpal boss and osseous coalition: an anatomic study. J Hand Surg Am 2009;34:1–6. 10.1016/j.jhsa.2008.08.025 19081681

[3] Clarke AM , Wheen DJ, Visvanathan S, et al. The symptomatic carpal boss: is simple excision enough? J Hand Surg 1999;24:591–5. 10.1054/JHSB.1999.0238 10597939

[4] Park MJ , Namdari S, Weiss AP. The carpal boss: review of diagnosis and treatment. J Hand Surg Am 2008;33:446–9. 10.1016/j.jhsa.2007.11.029 18343306

[5] Conway WF , Destouet JM, Gilula LA, Bellinghausen HW, Weeks PM. The carpal boss: an overview of radiographic evaluation. Radiology 1985;156:29–31. 10.1148/radiology.156.1.3923555 3923555

[6] Kissel P . Conservative management of symptomatic carpal bossing in an elite hockey player: a case report. J Can Chiropr Assoc 2009;53:282–9. 20037693 PMC2796947

[7] Agha RA , Franchi T, Sohrabi C, et al. Guideline: updating consensus surgical CAse REport (SCARE) guidelines. Int J Surg 2020;84:226–30. 10.1016/j.ijsu.2020.10.034 33181358

[8] Zanetti M , Saupe N, Nagy L. Role of MR imaging in chronic wrist pain. Eur Radiol 2007;38;17:927. 16932876 10.1007/s00330-006-0365-4

[9] Porrino J , Maloney E, Chew FS. Current concepts of the carpal boss: pathophysiology, symptoms, clinical or imaging diagnosis, and management. Curr Probl Diagn Radiol 2015;44:462–8. 10.1067/j.cpradiol.2015.02.008 25858555

[10] duBrutz LD . Carpal boss and the differential diagnosis of dorsal hand masses. J Am Board Fam Pract 1994;7:248–9. 8059631

[11] Karmazyn B , Siddiqui AR. Painful os styloideum in a child. Pediatr Radiol 2002;32:370–2. 10.1007/s00247-001-0639-6 11956728

[12] Keupers M , Gelin G, Vandevenne J, Grieten M. Carpal boss syndrome. JBR-BTR 2012;95:1–7. 10.5334/jbr-btr.678 23198375

[13] Capo JT , Orillaza NS, Lim PK. Carpal boss in an adolescent: case report. J Hand Surg Am 2009;34:1808–10. 10.1016/j.jhsa.2009.07.022 19897321

[14] Fusi S , Watson HK, Cuono CB. The carpal boss: a 20-year review of operative management. J Hand Surg 1995;20:405–8. 10.1016/S0266-7681(05)80104-4 7561423

[15] Cuono CB , Watson HK. The carpal boss: surgical treatment and etiological considerations. Plast Reconstr Surg 1979;63:88–93. 10.1097/00006534-197901000-00014 432327

